# Drying Method‐Dependent Variations in Phenolics, Color, and Volatile Profile of *Thymus praecox* subsp. *skorpilii*

**DOI:** 10.1111/1750-3841.71253

**Published:** 2026-06-30

**Authors:** Didem Karadeniz, Işıl Barutçu Mazı, Öznur Ergen Akçin

**Affiliations:** ^1^ Department of Molecular Biology and Genetics Ordu University Ordu Turkey; ^2^ Department of Food Engineering Ordu University Ordu Turkey

**Keywords:** color parameters, drying methods, headspace solid‐phase microextraction coupled with gas chromatography–mass spectrometry (HS‐SPME/GC–MS), headspace volatiles, microwave drying

## Abstract

This study investigated the effects of different drying methods, including freeze drying, oven drying, shade drying, and microwave drying (360, 600, and 900 W), on the volatile profile, total phenolic content (TPC), and color characteristics of wild‐grown *Thymus praecox* subsp*. skorpilii*. Headspace solid‐phase microextraction coupled with gas chromatography–mass spectrometry (HS‐SPME/GC–MS) analysis identified 26 volatile compounds in the fresh sample, with α‐pinene, 3‐octanone, limonene, and β‐caryophyllene as the predominant constituents. Drying induced pronounced shifts in volatile composition, characterized by an overall increase in isoprenoid compounds and a reduction in fatty acid derivatives, alongside the emergence of compounds such as nonanal and hexadecane. Increasing microwave power led to a decrease in monoterpenes and an increase in sesquiterpenes. TPC (19.5–40.21 mg gallic acid equivalent [GAE]/g), and color parameters were also significantly affected (*p* < 0.05), with freeze drying and high‐power microwave drying showing improved phenolic retention and color preservation compared to oven and shade drying. Overall, the results indicate that drying influences multiple quality attributes in a method‐dependent manner, with variations in processing conditions leading to distinct compositional outcomes. These findings highlight the importance of aligning drying conditions with specific quality targets when processing wild thyme.

**Practical Applications:**

This study establishes a practical framework for selecting drying strategies in wild thyme (*Thymus praecox* subsp. *skorpilii*) by linking processing conditions to changes in volatile composition, color, and phenolic content. The results demonstrate that drying induces method‐dependent transformations rather than uniform quality changes. Accordingly, drying methods should be chosen on the basis of the targeted product profile together with processing efficiency, enabling more tailored production of high‐quality dried thyme.

## 1 Introduction

Aromatic and medicinal plants have long held significant roles in traditional medicine, culinary applications, and the pharmaceutical and cosmetic industries due to their rich bioactive composition (Anuradha and Bharadvaja 2023). Among these, the genus *Thymus* (Lamiaceae) is particularly valued for its diverse phytochemical profile and associated biological activities, including antioxidant, antimicrobial, and anti‐inflammatory effects (Waheed et al. 2024; Jalil et al. 2024; Anwar et al. 2024; Li et al. 2019). In Turkey, the genus is represented by 42 species, 48% of which are endemic (Celep and Dirmenci 2017).

*Thymus praecox* Opiz, commonly known as “plateau thyme,” is naturally distributed across the northern regions of Turkey, particularly in high‐altitude habitats (Uzun et al. 2023). According to Jalas (1980), *T. praecox* is represented in Turkey by two subspecies: subsp. *skorpilii* (Velen.) Jalas (syn. subsp. *jankae* [Celak.]), and subsp. *grossheimii* (Ronniger) Jalas. *T. praecox* subsp. *skorpilii* is a low‐growing, creeping, woody‐based perennial herbaceous plant. The basal leaves are typically sessile, whereas the stem leaves are at least twice as long as they are wide. The stem, leaves, and calyx are covered with numerous trichomes and glandular hairs, giving the plant a characteristic spicy aroma. The flowers are mauve‐purple, and the species is commonly found on rocky and stony mountain slopes.

Previous studies on this taxon have primarily focused on its essential oil composition and bioactivity. For instance, Baser et al. (1996) investigated the essential oil composition of different *T. praecox* varieties collected from Turkey, reporting that major constituents varied depending on the taxonomic unit studied. Similarly, Avcı (2011) examined the essential oil of this subspecies across different altitudinal populations in the southern part of Turkey and demonstrated that dominant compounds, such as thymol and carvacrol, showed significant variation depending on location. In another study, Ozen et al. (2011) evaluated samples collected from the Black Sea region of Turkey and identified thymol and *o*‐cymene as major constituents of the essential oil, alongside notable antioxidant activity. Furthermore, studies focusing on headspace volatiles have also revealed considerable variation among closely related taxa. Stojanović et al. (2014) reported that *T. praecox* subsp. *jankae* (syn. subsp. *skorpilii*), from Serbian and Bulgarian populations was dominated by monoterpene hydrocarbons such as α‐pinene, myrcene, limonene, and β‐pinene, whereas Vidic et al. (2010) identified linalyl acetate as the predominant compound in this subspecies, followed by α‐pinene. Collectively, these findings demonstrate that *T. praecox* subsp. *skorpilii* exhibits considerable chemical variability, which is largely influenced by genetic background, geographical origin, and environmental conditions (Uzun et al. 2024; Stojanović et al. 2014; Avcı 2011; Vidic et al. 2010; Baser et al. 1996). Despite this well‐documented compositional variability, there is still a lack of systematic studies evaluating how different drying methods influence the volatile profile and quality attributes of this subspecies.

Drying is a critical post‐harvest process for aromatic and medicinal plants, aiming to reduce moisture content, inhibit microbial growth, and stabilize phytochemicals during storage and further processing. Numerous studies have shown that drying can cause both qualitative and quantitative changes in volatile organic compounds (VOCs), leading to the degradation, loss, or even formation of new compounds (Nurhaslina et al. 2022; Chua et al. 2019; Sárosi et al. 2013). In addition, color parameters and total phenolic content (TPC), critical indicators of dried herb quality, are also known to vary depending on the drying technique (Nurhaslina et al. 2022). Headspace solid‐phase microextraction coupled with gas chromatography–mass spectrometry (HS‐SPME/GC–MS) has become one of the most widely used analytical approaches for investigating volatile composition and relative changes in plant‐derived volatiles due to its solvent‐free nature, high sensitivity, and suitability for comparative profiling studies. Recent studies have successfully employed this technique to evaluate volatile profiles and compositional variations in aromatic and medicinal plants under different biological and processing conditions (Guo 2026; Ergun et al. 2026).

A wide range of drying methods, including freeze drying, oven drying, vacuum drying, shade and sun drying, solar‐assisted drying, and microwave drying, have been applied to different *Thymus* species, and their effects on chemical composition and quality attributes have been extensively reported (Yilmaz et al. 2021; Chua et al. 2019; Rahimmalek and Goli 2013; Sárosi et al. 2013). However, the outcomes of these studies are not consistent across species. For instance, drying has been reported to increase thymol content in some species such as *Thymus daenensis* (Dehghani Mashkani et al. 2018), whereas in others, including *Thymus vulgaris*, its concentration may decrease (Alqarni et al. 2022) or vary depending on the drying method applied (Rahimmalek and Goli 2013; Sárosi et al. 2013). Similarly, certain compounds may be lost during drying, whereas others may be newly formed, and these patterns vary not only between species but also with the drying technique, even within the same species (Rahimmalek and Goli 2013; Sárosi et al. 2013; D'Auria and Racioppi 2015; Alqarni et al. 2022).

These findings suggest that the impact of drying is not universal but rather species‐dependent, and therefore cannot be generalized across the genus *Thymus*. Despite the increasing number of studies on cultivated and commercially available species, investigations focusing on wild‐collected taxa remain limited. Consequently, it remains unclear how drying processes influence the volatile profile and overall quality attributes of less‐studied species such as *T. praecox* subsp. *skorpilii*.

In this context, the present study aims to systematically evaluate the effects of different drying methods, including microwave drying at varying power levels, oven drying, freeze drying, and shade drying, on the volatile composition, TPC, and color properties of *T. praecox* subsp. *skorpilii*. By integrating chemical and quality parameters, this study provides a comprehensive assessment of drying‐induced changes in a wild‐growing and phytochemically variable subspecies, thereby contributing to a better understanding of species‐specific responses to drying.

## 2 Materials and Methods

### 2.1 Chemicals and Reagents

All chemicals and reagents used in this study were of analytical grade. Methanol (≥99.8%), Folin‐Ciocalteu reagent (2N), sodium carbonate (≥99.5%), gallic acid (≥98.0%), and *n*‐alkane standard mixture (C7–C30) were obtained from Sigma‐Aldrich Chemical Co. Distilled water was used throughout the experiments. Helium gas (99.999% purity) was supplied by a local gas provider and used as carrier gas in GC–MS analysis. A CAR/PDMS fiber (75 µm) for SPME was obtained from Supelco (Germany).

### 2.2 Plant Material

**FIGURE 1:**
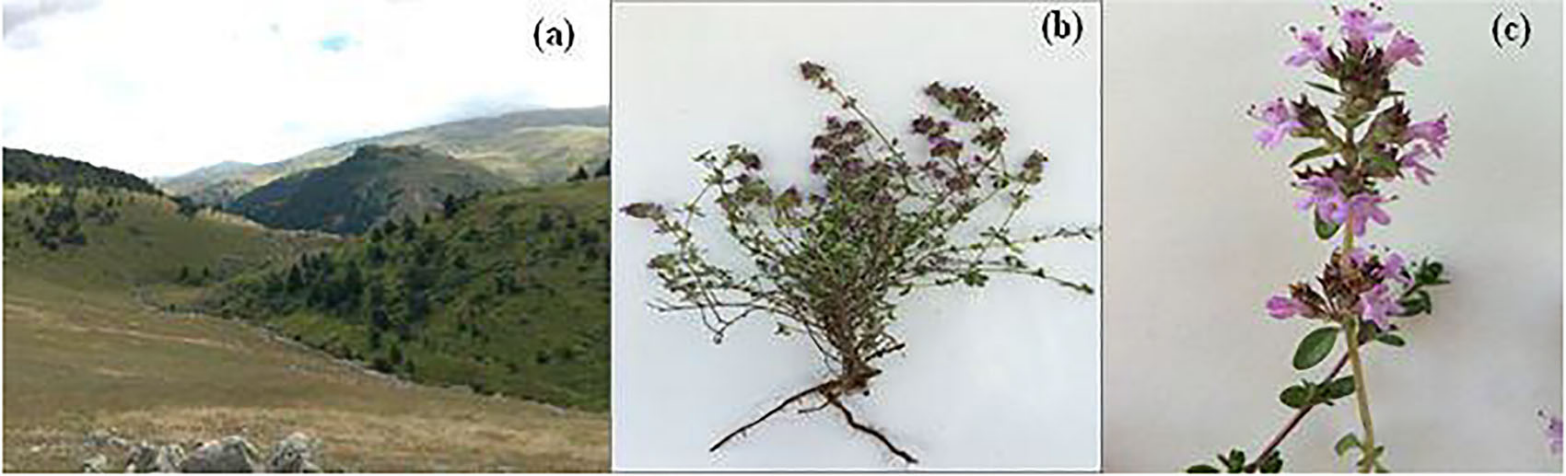
Geographical location of the *Thymus praecox* subsp. *skorpilii* collection site (a), whole plant morphology, including root and stem (b), and close‐up view of the inflorescence during the flowering stage (c).

*T. praecox* subsp. *skorpilii* specimens were collected during the flowering stage in August 2023 from the Çambaşı‐Eskibağlak Plateau located in the highlands of Ordu Province, northeastern Turkey (Figure 1). The collection site is situated at an altitude of approximately 1850 m (40°37′38″ N, 37°57′57″ E). Voucher specimens were identified by Dr. Öznur Ergen Akçin and deposited at the Botany Laboratory, Faculty of Arts and Sciences, Ordu University, under voucher number “Karadeniz‐103.”

### 2.3 Sample Preparation

The leaves and stems of the plant samples were separated on the day of harvest. They were stored in sealed plastic bags at +4°C overnight and dried the following day using different drying techniques.

### 2.4 Drying Methods

Drying was performed using four different techniques, including microwave drying, oven drying, freeze drying, and shade drying. The initial moisture content of the samples was determined using the standard oven drying method at 105°C until constant weight. Preliminary trials were conducted for each drying method, during which moisture loss was monitored gravimetrically at predefined time intervals. On the basis of these trials, the drying durations required to reach a final moisture content below 10% (w.b.) were determined and subsequently applied in the main experiments. For shade drying, the process was continued under ambient conditions until the sample weight stabilized. The selection of a final moisture content of approximately 10% (w.b.) was based on commonly reported values for aromatic and medicinal plants in the literature (Dehghani Mashkani et al. 2018; Rodríguez et al. 2014). All experiments were conducted in triplicate.

#### 2.4.1 Microwave Drying

Microwave drying was performed using a household microwave oven (Samsung Smart Oven, MC32F606) at power levels of 360, 600, and 900 W, representing low, medium, and high settings of the device. These levels were selected to cover a representative range of microwave intensities within the 180–1000 W interval reported in the literature for thyme drying (Yilmaz et al. 2021; Rahimmalek and Goli 2013), while also corresponding to the discrete power settings available in the microwave oven. Approximately 5.00 ± 0.01 g of the sample was placed in a Petri dish (11.5 cm diameter) and positioned at the center of the turntable.

#### 2.4.2 Oven Drying

Oven drying was conducted in a laboratory‐scale fan‐assisted oven (NST‐55, Nukleon, Turkey) at 50°C, a temperature selected on the basis of literature data (Sárosi et al. 2013; Rahimmalek and Goli 2013) and preliminary trials. For each run, approximately 5.00 ± 0.01 g of the sample was placed in a Petri dish and positioned at the center of the oven.

#### 2.4.3 Freeze Drying

Freeze drying was conducted in a laboratory‐scale lyophilizer (FreeZone 2.5 L, 7670530, Labconco) at −50°C and 0.1 mbar vacuum pressure. The same sample quantity and preparation were used to ensure consistency across methods. Drying time was set at 120 h on the basis of preliminary trials to obtain a final moisture content below 10% (w.b.).

#### 2.4.4 Shade Drying

Shade drying was carried out in a well‐ventilated, shaded environment at an average temperature of 29.4°C and relative humidity of 70.2%, representing the ambient conditions during the drying process, which were monitored throughout the experiment. Plant material was spread in thin layers on trays, with a layer thickness of approximately 8 mm, similar to that of the samples placed in Petri dishes for the other methods. The process lasted for 72 h, after which the samples reached a final moisture content of approximately 12% (w.b.), as further moisture removal was limited under ambient conditions.

### 2.5 Moisture Content Analysis

Moisture content was determined gravimetrically by drying the samples at 105°C until constant weight of the sample.

### 2.6 TPC Analysis

A 0.5‐g portion of dried sample was mixed with 80% methanol (1:10 ratio), vortexed for 2 min (Isolab, Germany), and extracted in an ultrasonic bath (28 kHz, 500 W; CleanEx 411, Everest Ultrasonic, Turkey) at 30°C for 15 min. The mixture was centrifuged at 10,000 × *g* for 5 min (D2012 Plus, Isolab, Germany), and the supernatant was collected. TPC was measured using a modified Folin‐Ciocalteu method (Slinkard and Singleton 1977). Briefly, 60 µL of supernatant was mixed with 3.48 mL of distilled water and 0.300 mL of Folin‐Ciocalteu reagent (2N, used as supplied without dilution), followed by 0.900 mL of 20% Na_2_CO_3_ solution. The mixture was vortexed and incubated at 40°C for 30 min (WiseBath/Wsb‐18, Daihan Scientific, Korea). Absorbance was measured at 760 nm using a UV spectrophotometer (UV mini‐1240, Shimadzu, Japan). A calibration curve was constructed using gallic acid standard solutions in the range of 20–100 mg/L, and the results were expressed as mg gallic acid equivalents (GAE) per g dry matter (DM) (Yilmaz et al. 2019).

### 2.7 Volatile Compound Analysis (HS‐SPME/GC–MS)

The analysis of volatile compounds was carried out using HS‐SPME/GC–MS. Precisely weighed samples (1.05–2.00 g) were transferred into 15 mL headspace vials, ensuring that approximately one‐third of the vial volume was occupied. The vials were hermetically sealed and subjected to incubation at 60°C for 15 min in the absence of a fiber, followed by 30 min in the presence of a CAR/PDMS fiber (75 µm, Supelco, Germany) for volatile compound extraction. Subsequently, the fiber was thermally desorbed in the injection port of a GC–MS system (GCMS‐QP2010 PLUS, Shimadzu, Japan) equipped with an Rxi‐5MS capillary column (30 m × 0.25 mm × 0.25 µm, Restek, USA). The gas chromatograph (GC) oven temperature was initially held at 40°C for 2 min then programmed to increase at a rate of 4°C/min to a final temperature of 250°C, which was maintained for an additional 5 min. The injector and mass spectrometric detector temperatures were both set to 250°C. Helium was employed as the carrier gas at a constant flow rate of 1.61 mL/min, with EI (70 eV) as the ionization method. Volatile compounds were identified by comparing their mass spectra with those in the Wiley, NIST, and FFNSC spectral libraries. Linear retention indices (LRIs) were determined by analyzing a C7–C30 *n*‐alkane standard mixture (Sigma‐Aldrich, USA) under the same chromatographic conditions.

The identification of volatile compounds was based on comparison of mass spectra with commercial libraries (NIST, Wiley, and FFNSC) and by comparing experimentally calculated LRIs with literature values. LRIs were determined relative to a homologous series of *n*‐alkanes (C7–C30) using the equation of Van den Dool and Kratz (1963).

### 2.8 Color Measurement

The L*, a*, and b* color parameters of the dried samples were measured using a chromameter (Minolta CR‐400, Minolta Co. Ltd., Osaka, Japan). L* represents lightness (ranging from black to white), a* indicates the green–red axis, and b* reflects the blue–yellow axis. Measurements were performed in triplicate from three different areas of each sample, and average values were reported.

### 2.9 Statistical Analysis

Data were analyzed using one‐way analysis of variance (ANOVA) to assess the effect of different drying methods. When significant differences were detected (*p *≤ 0.05), Tukey's multiple comparison test was applied to determine specific group differences. Principal component analysis (PCA) was also conducted to explore the multivariate patterns of VOC composition across treatments. All statistical analyses, including PCA, were performed using MINITAB 17 software.

## 3 Results and Discussion

### 3.1 Drying Time and Moisture Content

The moisture content of the fresh thyme sample used in this study was determined to be 64.2% (w.b.). Drying durations required to reach the target moisture content (≈10% w.b.) varied markedly depending on the applied method (Table 1). Microwave drying achieved this level within 2.0–4.5 min, whereas oven drying required 6 h and shade drying required 72 h. Freeze drying, although highly effective, required the longest processing time (120 h). All drying methods, except shade drying, reduced the moisture content to the target level, whereas shade drying remained slightly higher (≈12%). This can be attributed to the inherent limitations of shade drying, which depends strongly on ambient conditions; in this study, the relatively high relative humidity (70.2%) and moderate temperature (29.4°C) reduced the drying driving force, leading to a substantial reduction in drying rate as equilibrium moisture conditions were approached. The substantially shorter drying time observed in microwave treatments compared to conventional methods is primarily due to rapid internal heat generation, which accelerates moisture evaporation and transport (Zhang et al. 2006). In contrast, oven and shade drying rely on external heat transfer, resulting in slower moisture removal, particularly at later stages (Chua et al. 2019). As expected, increasing microwave power further reduced drying time. Drying at 900 W shortened the process by approximately 55% compared to 360 W. Similar trends have been reported in previous studies on thyme and other aromatic herbs (Yilmaz et al. 2021; Chua et al. 2019). Water activity (*a_w_*) values ranged from 0.18 (freeze‐dried) to 0.51 (shade‐dried), whereas the remaining drying methods showed relatively similar values within this interval (Table 1). Despite these differences, all samples exhibited *a_w_* values below the critical threshold of 0.60, which is widely accepted as the limit for inhibiting microbial growth in dried foods (Lopez‐Malo and Alzamora 2015). The higher *a_w_* observed in shade drying can be attributed to limited moisture removal under ambient conditions; however, it still remained within a range considered microbiologically stable.

**TABLE 1:** Drying durations and final moisture contents of *Thymus praecox* ssp. *skorpilii* samples.

Drying method	Duration	Moisture content (%)^*^	Water activity (*a_w_*)
Freeze drying	120 h	3.05^c^ ± 0.88	0.18^c^ ± 0.01
Oven drying	6 h	8.92^b^ ± 0.19	0.40^b^ ± 0.00
Shade drying	72 h	12.1^a^ ± 1.15	0.51^a^ ± 0.01
Microwave drying (360 W)	4.5 min	7.94^b^ ± 0.70	0.38^b^ ± 0.02
Microwave drying (600 W)	2.5 min	6.49^b^ ± 0.65	0.36^b^ ± 0.01
Microwave drying (900 W)	2.0 min	7.93^b^ ± 0.79	0.39^b^ ± 0.01

*Note*: Different letters in each column show a significant difference (*p *< 0.05) between means.

^*^Moisture contents are expressed on a wet basis (% w.b.).

### 3.2 Color

Color is one of the key quality parameters in dried herbal products, reflecting both processing conditions and consumer appeal. The CIELAB parameters (L*, a*, and b*) were used to evaluate color changes induced by different drying methods. The fresh sample exhibited L*, a*, and b* values of 31.56, −2.50, and 9.88, respectively, with the negative a* value confirming the dominance of green tones.

**FIGURE 2:**
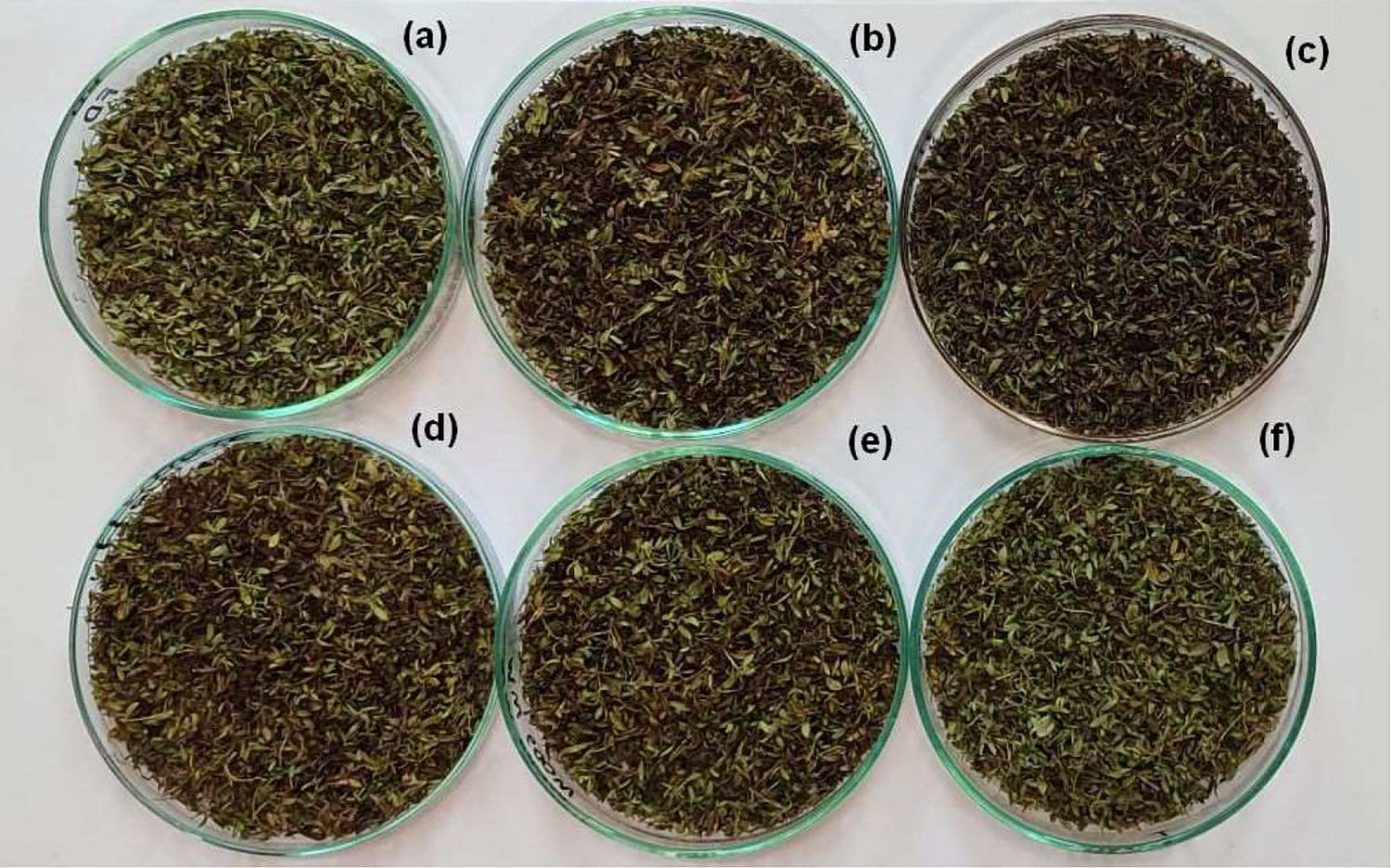
Visual appearance of *Thymus praecox* subsp. *skorpilii* samples dried by different methods: freeze‐dried (a), oven‐dried (b), shade‐dried (c) and microwave‐dried at 360 W (d), 600 W (e), and 900 W (f).

Drying significantly influenced color parameters (*p *< 0.05), as also illustrated in Figure 2. Following drying, L* values ranged from 26.63 to 33.49, a* values from −2.50 to 1.41, and b* values from 5.51 to 9.75 (Table 2). Among the methods, freeze drying and microwave drying better preserved lightness and green coloration, whereas oven and shade drying resulted in darker samples with higher a* values, indicating a shift toward reddish–brown tones. Consistent with the present findings, previous studies have demonstrated that drying generally results in decreased L* values and increased a* values in various thyme species (Yilmaz et al. 2021; Rahimmalek and Goli 2013).

**TABLE 2:** Color parameters of fresh and dried *Thymus praecox* ssp. *skorpilii* samples.

Sample	L*	a*	b*
Fresh	31.55^ab^ ± 2.50	−2.50^c^ ± 1.91	9.88^a^ ± 1.82
Freeze‐dried	33.49^a^ ± 3.46	−1.82^c^ ± 1.41	9.75^a^ ± 2.11
Oven‐dried	28.49^bc^ ± 2.77	−0.01^ab^ ± 1.23	6.09^bc^ ± 2.37
Shade‐dried	26.63^c^ ± 2.79	1.41^a^ ± 0.99	5.51^c^ ± 1.78
Microwave‐dried (360 W)	30.64^ab^ ± 2.60	−1.52^bc^ ± 1.30	8.82^ab^ ± 2.41
Microwave‐dried (600 W)	30.90^ab^ ± 3.06	−1.52^bc^ ± 1.84	9.04^a^ ± 2.67
Microwave‐dried (900 W)	31.37^ab^ ± 3.85	−2.09^c^ ± 1.83	9.36^a^ ± 3.29

*Note*: Different letters in each column show a significant difference (*p* < 0.05) between means.

The green coloration of thyme is determined by chlorophyll pigments, particularly chlorophyll *a*, whereas carotenoids contribute yellow tones but are typically masked (El‐Qudah 2014). The observed increase in a* values is closely associated with chlorophyll degradation, which occurs under the influence of heat, light, and oxygen, as well as enzymatic activity (Tonucci and Von Elbe 1992; Orphanides et al. 2016). In addition, non‐enzymatic browning reactions, including Maillard reactions, contribute to color changes during drying (Pathare et al. 2013). Maillard reactions involve interactions between reducing sugars and amino compounds, leading to the formation of brown‐colored melanoidins, which contribute to sample darkening (lower L*) and a shift toward red‐brown tones (higher a*), while also potentially masking yellow pigments and thereby affecting b* values (Weemaes et al. 1999). The extent of these changes depends strongly on drying temperature and duration. Accordingly, prolonged drying enhances pigment degradation, particularly in shade drying due to enzymatic and oxidative processes, whereas oven drying may also promote non‐enzymatic browning, resulting in higher a* and lower L* values. In contrast, freeze drying minimizes these effects due to low temperature and reduced oxygen availability, thereby preserving pigment integrity (Chua et al. 2019). Microwave drying also resulted in lower a* and higher L* values, indicating improved color retention. This may be explained by shorter processing time, which limits exposure to conditions that promote pigment degradation and Maillard browning. In addition, the better preservation of green color at higher microwave power levels may be attributed to rapid internal heating, which leads to faster temperature increases and promotes the inactivation of chlorophyll‐degrading enzymes such as chlorophyllase, thereby limiting chlorophyll degradation (Barutçu Mazı and San 2023; Orphanides et al. 2016). This is supported by Yilmaz et al. (2021), who reported higher chlorophyll retention in thyme samples dried at higher microwave power levels. The b* values showed a slight decrease after drying, although this change was significant mainly in oven‐ and shade‐dried samples. In contrast, freeze‐ and microwave‐dried samples exhibited relatively stable b* values, indicating a better preservation of yellow pigments. The variability reported in the literature, even for similar drying methods, suggests that thyme species and drying conditions play a critical role in determining color changes (Yilmaz et al. 2021; Rahimmalek and Goli 2013).

### 3.3 Total Phenolic Content

**FIGURE 3:**
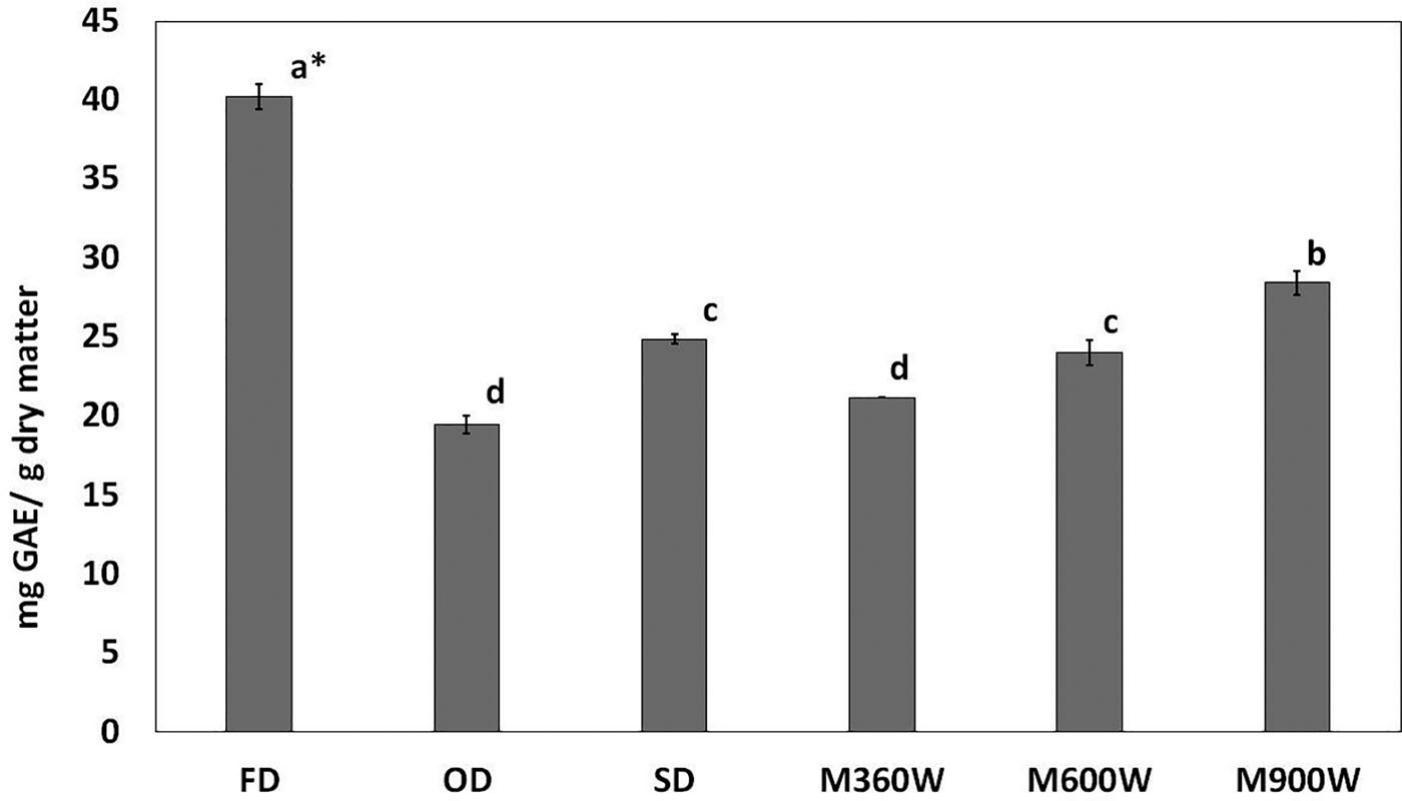
Total phenolic content of dried *Thymus praecox* ssp. *skorpilii* samples. *Different letters in each column show a significant difference (*p* < 0.05) between means. FD, freeze‐dried; GAE, gallic acid equivalents; M360W, microwave‐dried at 360 W; M600W, microwave‐dried at 600 W; M900W, microwave‐dried at 900 W; OD, oven‐dried; SD, shade‐dried.

The TPC of the dried thyme samples ranged from 19.5 to 40.2 mg GAE/g DM (Figure 3). Studies specifically focusing on *T. praecox* subsp. *skorpilii* are limited and reported values range from approximately 2.8 to 70 mg GAE/g depending on extraction methods and growing conditions (Taşkın et al. 2019; Ozen et al. 2011). The values obtained in the present study fall within this reported range.

Drying method had a significant effect on TPC (*p *< 0.05). Freeze drying yielded the highest phenolic retention, confirming its effectiveness in preserving thermolabile compounds. Shade drying resulted in moderate TPC values. Although low temperatures may help preserve heat‐sensitive compounds, the prolonged drying time allows oxidative enzymes to remain active for longer periods, leading to partial degradation of phenolics (Chua et al. 2019). Among drying treatments, microwave drying at 900 W yielded the second‐highest TPC, whereas lower power levels resulted in reduced values. The higher TPC at 900 W can be explained by the increased rate of energy input, leading to rapid internal heating and the development of internal pressure, which facilitates cell wall disruption and the release of bound phenolics (Chua et al. 2019; Hamrouni‐Sellami et al. 2013). Moreover, the elevated temperatures at this power level may enhance the inactivation of oxidative enzymes, such as polyphenol oxidase, thereby limiting phenolic degradation. Although elevated temperatures may adversely affect heat‐sensitive compounds, the shorter exposure time at higher power levels may partly offset this effect. Even at similar drying durations, internal temperature rise varies markedly with microwave power (Barutçu Mazı and San 2023). At 360 W, the lower energy input may not be sufficient to induce significant cell rupture or to effectively inactivate oxidative enzymes. However, compared to hot‐air drying, the shorter drying duration may limit enzymatic degradation of phenolics. This balance may explain why microwave drying at 360 W resulted in TPC values comparable to, or slightly higher than, those obtained by oven drying. Consistent with our findings, Hamrouni‐Sellami et al. (2013) reported a positive correlation between increasing microwave power and TPC in sage. However, contrasting trends have also been reported in the literature. Tohidi‐Nejad et al. (2024) showed that the effect of drying method on thyme is strongly influenced by seasonal variation: in some cases, 360 W microwave drying yielded the highest TPC, whereas in others oven drying produced comparable or even higher values. Yilmaz et al. (2021) found that increasing microwave power from 200 to 1000 W led to a gradual decrease in TPC, with values at 1000 W comparable to those obtained by hot‐air drying at 50°C. Collectively, these findings indicate that phenolic responses to drying are highly matrix‐ and context‐dependent rather than following a universal trend. The results of the present study confirm a strong influence of drying conditions on the phenolic content of *T. praecox* subsp. *skorpilii*. Taken together, these findings indicate that phenolic responses to drying are governed by interacting factors such as thermal exposure, drying duration, and matrix disruption, rather than a single dominant variable, suggesting method‐dependent variation patterns across conditions.

### 3.4 Volatile Compound Profile

HS‐SPME, a simple and solvent‐free green technology, was employed to analyze the VOCs in *T. praecox* subsp. *skorpilii*. VOCs in plants are generally classified into seven major chemical groups: benzenoids, phenylpropanoids, fatty acid derivatives, isoprenoids, nitrogen‐ and sulfur‐containing compounds, and others (including heterocyclic compounds, lactones, spiroacetals, and naphthalenes) (Knudsen et al. 1993). In the fresh sample of *T. praecox* subsp. *skorpilii*, a total of 26 VOCs were identified (Table 3). These compounds were predominantly composed of isoprenoids, which accounted for 73.88% of the total VOC content, with monoterpene hydrocarbons, sesquiterpene hydrocarbons, and oxygenated monoterpenes contributing 52.08%, 20.37%, and 1.43%, respectively. Monoterpenoids represented the largest VOC class. The most abundant compounds detected were α‐pinene (15.63%), 3‐octanone (14.76%), limonene (13.47%), β‐caryophyllene (8.41%), and valencene (7.07%). Both α‐pinene and limonene are monoterpene hydrocarbons commonly found in the essential oils of many aromatic plants. These compounds are known not only for their distinctive aromas but also for their broad biological activities, including immunomodulatory, anti‐inflammatory, anticancer, and antioxidant properties (Masyita et al. 2022). In addition, they hold promising potential as natural food preservatives for a variety of food products (Masyita et al. 2022). Besides isoprenoids, fatty acid derivatives were also markedly present in the fresh sample, accounting for 19.86% of the total VOCs. Among these, 3‐octanone (14.76%) was the most dominant, followed by 1‐octen‐3‐ol (3.72%) and 3‐octanol (1.38%). These C8 volatiles are well documented in fungi but are less frequently reported in plants, where they serve as defensive compounds, particularly in species from the Lamiaceae and Fabaceae families (Pennerman et al. 2022). Several headspace‐based studies have investigated the volatile composition of *T. praecox* subsp. *skorpilii* in different regions, primarily within the Balkans and northwestern Turkey. In a study conducted on leaves and flowers of *Thymus jankae* Celak (Syn: *T. praecox* subsp. *skorpilii*) collected from high‐altitude sites in the Bursa region of Turkey (1800–2200 m), thymol (17.65%), *p*‐cymene (15.34%), carvacrol (13.27%), and γ‐terpinene (10.73%) were identified as the dominant compounds (Uzun et al. 2024). In samples from Bosnia and Herzegovina, linalyl acetate (52.4%) and α‐pinene (14.5%) were found to be the main constituents in the aerial parts of wild‐growing *T. praecox* subsp. *skorpilii* (Vidic et al. 2010). These findings highlight considerable variability in the main headspace volatiles of *T. praecox* subsp. skorpilii. Stojanović et al. (2014), analyzing above‐ground parts of *T. praecox* subsp. *jankae* from Serbia and Bulgaria, reported α‐pinene (29.4% and 18.6%), myrcene (12.1% and 23.2%), limonene (7.5% and 17.8%), and β‐pinene (11.7% and 7.6%) as the major volatiles in those respective samples. Notably, thymol, a phenolic monoterpenoid, was not detected in our samples, nor was it identified in the headspace compositions of the Bosnian (Vidic et al. 2010) and Bulgarian (Stojanović et al. 2014), samples. In some of these earlier studies, fatty acid derivatives were also detected in *T. praecox* taxa, albeit in lower proportions compared to our findings. Literature underscores the influence of ecological and geographical variation, as well as differences in harvest time and phenological stage, on the VOC composition of *T. praecox* taxa (Avcı 2011; Baser et al. 1996; Ozen et al. 2011). Moreover, unlike previous research that generally analyzed combined leaf and flower tissues, our study focused exclusively on leaves and stems, which may have contributed to the observed differences in VOC profiles.

**TABLE 3:** Headspace volatile constituents (area %) identified in fresh and dried *Thymus praecox* subsp. *skorpilii* samples.

LRI^a^	Components	Group	Fresh	Freeze‐dried	Shade‐dried	Oven‐dried	Microwave‐dried (W)		
							360	600	900
925	α‐Thujene	Monoterpene	1.41 ± 0.14	0.94 ± 0.01	0.80 ± 0.01	0.57 ± 0.03	1.61 ± 0.03	1.78 ± 0.07	1.63 ± 0.06
931	α‐Pinene	Monoterpene	15.63 ± 0.34	9.62 ± 0.86	10.14 ± 0.04	7.37 ± 0.27	18.04 ± 2.36	17.51 ± 0.16	14.67 ± 0.16
945	Camphene	Monoterpene	6.53 ± 0.06	3.20 ± 0.33	3.84 ± 0.06	2.61 ± 0.07	5.50 ± 0.06	5.57 ± 0.45	4.58 ± 0.06
956	Benzaldehyde	Benzenoid	0.24 ± 0.01	0.30 ± 0.03	1.28 ± 0.11	0.78 ± 0.07	0.50 ± 0.03	0.36 ± 0.13	0.20 ± 0.04
971	Sabinene	Monoterpene	0.31 ± 0.11	0.30 ± 0.03	0.32 ± 0.00	0.15 ± 0.01	0.41 ± 0.03	0.52 ± 0.03	0.33 ± 0.04
973	β‐Pinene	Monoterpene	0.85 ± 0.01	0.44 ± 0.03	0.53 ± 0.03	0.38 ± 0.01	0.75 ± 0.06	0.73 ± 0.03	0.52 ± 0.01
979	1‐Octen‐3‐ol	Fatty acid derivatives	3.72 ± 0.20	2.83 ± 0.20	0.53 ± 0.01	0.71 ± 0.03	0.20 ± 0.01	0.21 ± 0.01	0.08 ± 0.00
986	3‐Octanone	Fatty acid derivatives	14.76 ± 0.51	1.50 ± 0.20	1.78 ± 0.03	1.11 ± 0.06	0.45 ± 0.01	0.38 ± 0.07	0.42 ± 0.03
990	β‐Myrcene	Monoterpene	6.14 ± 1.26	4.58 ± 0.24	4.25 ± 0.06	3.56 ± 0.34	6.59 ± 0.28	8.15 ± 0.04	8.70 ± 0.18
995	3‐Octanol	Fatty acid derivatives	1.38 ± 0.49	1.07 ± 0.14	0.81 ± 0.03	0.64 ± 0.07	0.43 ± 0.01	0.49 ± 0.01	0.52 ± 0.04
1002	α‐Phellandrene	Monoterpene	0.16 ± 0.01	0.42 ± 0.03	0.53 ± 0.00	0.28 ± 0.03	0.55 ± 0.04	0.71 ± 0.01	0.80 ± 0.01
1023	*p*‐Cymene	Monoterpene	4.31 ± 0.04	3.48 ± 0.37	3.29 ± 0.04	4.55 ± 0.07	4.02 ± 0.17	2.95 ± 1.03	2.66 ± 0.13
1027	Limonene	Monoterpene	13.47 ± 0.08	26.88 ± 4.38	21.81 ± 0.27	49.36 ± 1.13	26.36 ± 1.71	19.87 ± 0.27	19.20 ± 0.61
1048	β‐Ocimene	Monoterpene	2.31 ± 0.01	1.02 ± 0.31	1.31 ± 0.11	0.80 ± 0.04	1.18 ± 0.06	1.79 ± 0.04	1.64 ± 0.07
1057	γ‐Terpinene	Monoterpene	0.38 ± 0.00	0.49 ± 0.06	0.74 ± 0.00	0.75 ± 0.06	0.73 ± 0.04	0.70 ± 0.14	0.66 ± 0.06
1087	Terpinolene	Monoterpene	0.58 ± 0.01	0.75 ± 0.07	0.55 ± 0.00	0.53 ± 0.04	0.90 ± 0.06	0.59 ± 0.03	0.56 ± 0.01
1099	Linalool	Oxygenated monoterpenes	0.65 ± 0.00	3.43 ± 0.40	1.59 ± 0.01	1.07 ± 0.07	0.89 ± 0.03	0.69 ± 0.03	0.60 ± 0.01
1103	Nonanal	Fatty acid derivatives	n.d.	0.32 ± 0.06	1.62 ± 0.01	0.60 ± 0.03	0.52 ± 0.03	0.41 ± 0.03	0.29 ± 0.01
1143	Camphor	Oxygenated monoterpenes	0.78 ± 0.01	1.12 ± 0.11	0.96 ± 0.01	0.41 ± 0.01	0.73 ± 0.03	0.69 ± 0.01	0.38 ± 0.03
1388	β‐Bourbonene	Sesquiterpene	0.20 ± 0.00	0.73 ± 0.07	0.61 ± 0.13	0.42 ± 0.00	0.33 ± 0.01	0.34 ± 0.01	0.31 ± 0.01
1424	β‐Caryophyllene	Sesquiterpene	8.41 ± 0.62	22.24 ± 2.16	19.79 ± 0.23	10.58 ± 0.07	15.81 ± 0.30	19.22 ± 0.07	22.95 ± 0.14
1454	β‐Farnesene	Sesquiterpene	0.60 ± 0.01	0.24 ± 0.01	0.55 ± 0.04	0.22 ± 0.04	0.31 ± 0.01	0.30 ± 0.04	0.33 ± 0.04
1458	α‐Humulene	Sesquiterpene	1.63 ± 0.13	2.57 ± 0.28	2.39 ± 0.07	1.12 ± 0.01	1.86 ± 0.06	2.42 ± 0.01	3.25 ± 0.10
1485	Germacrene‐D	Sesquiterpene	0.64 ± 0.01	1.54 ± 0.21	1.27 ± 0.00	0.61 ± 0.08	0.75 ± 0.03	1.71 ± 0.06	1.70 ± 0.13
1499	Valencene	Sesquiterpene	7.07 ± 0.07	1.21 ± 0.13	2.74 ± 0.03	1.56 ± 0.06	1.39 ± 0.07	1.30 ± 0.00	1.89 ± 0.06
1519	γ‐Cadinene	Sesquiterpene	0.50 ± 0.01	0.64 ± 0.07	0.57 ± 0.00	0.25 ± 0.04	0.49 ± 0.00	0.84 ± 0.03	1.03 ± 0.14
1528	δ‐Cadinene	Sesquiterpene	1.33 ± 0.01	1.96 ± 0.24	1.91 ± 0.03	1.06 ± 0.03	1.76 ± 0.03	2.25 ± 0.31	2.79 ± 0.11
1590	Hexadecane	Others	n.d.	n.d.	4.49 ± 0.35	3.18 ± 0.18	1.01 ± 0.06	0.86 ± 0.03	0.77 ± 0.03

^a^Estimated linear retention indices (LRI) on Rxi‐5MS column.

**FIGURE 4:**
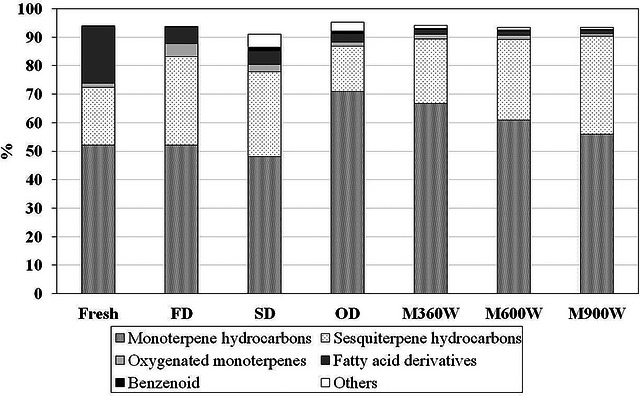
Grouped components of headspace volatile constituents (%) identified in fresh and dried *Thymus praecox* subsp. *skorpilii* samples. FD, freeze‐dried; M360W, microwave‐dried at 360 W; M600W, microwave‐dried at 600 W; M900W, microwave‐dried at 900 W; OD, oven‐dried; SD, shade‐dried.

Drying significantly influenced the headspace volatile composition of *T. praecox*. The number of identified VOCs increased slightly after drying, with 27 compounds detected in the freeze‐dried sample and 28 in all other dried samples (Table 3). Although the compound profile shifted depending on the method, three major volatiles, α‐pinene, limonene, and β‐caryophyllene, remained predominant across all dried samples, though their percentages varied. Drying had contrasting effects on two major classes of volatiles. Although the relative abundance of isoprenoids increased, fatty acid derivatives markedly decreased across all drying methods (Figure 4). This inverse pattern highlights a clear shift in the volatile profile of thyme following dehydration. Among isoprenoids, the proportions of several monoterpene and sesquiterpene hydrocarbons, including limonene, β‐caryophyllene, γ‐terpinene, β‐bourbonene, and α‐phellandrene, increased in all dried samples compared to the fresh material. In contrast, the concentrations of valencene, camphene, β‐ocimene, β‐pinene, and β‐farnesene were consistently lower after drying. Including α‐pinene, all remaining isoprenoids exhibited method‐dependent variations, with their levels either increasing or decreasing depending on the applied technique. Freeze drying maintained the percentage of total monoterpene hydrocarbons (TMH) at a level nearly identical to that in the fresh sample, whereas shade drying caused a slight reduction. Conversely, all other drying methods, particularly oven drying, resulted in increased monoterpene hydrocarbon levels. Monoterpene hydrocarbons may have decreased under shade drying due to the prolonged exposure time, which enhances evaporative and oxidative losses (Chua et al. 2019); in this study, the shade drying process lasted 72 h (Table 1). Regarding sesquiterpenes, oven drying led to a reduction in their relative content, whereas other techniques generally caused an increase.

These findings are consistent with literature reports, which show that depending on the herb type and drying conditions, both increases and decreases in the total content of monoterpene and sesquiterpene hydrocarbons have been reported, including in thyme (Chua et al. 2019; Sárosi et al. 2013). In the present study, drying resulted in an overall increase in the percentage of total isoprenoids. The smallest increase in total isoprenoid content was observed in the shade‐dried sample (8.95%), whereas the most pronounced enhancement occurred in microwave‐dried samples, with an increase of approximately 23%. On the basis of relative percentages, the three most abundant isoprenoids in the fresh sample, α‐pinene, limonene, and β‐caryophyllene, reached their respective maxima in the 360 W microwave, oven, and 900 W microwave‐dried samples, respectively. Although oven drying resulted in the highest relative percentage of limonene (49.36%), this increase may also reflect a proportional rise caused by the substantial reduction of other monoterpene and sesquiterpene hydrocarbons under the same treatment. Indeed, oven drying yielded the lowest levels of all other terpenoid hydrocarbons, except for valencene, β‐bourbonene, *p*‐cymene, and γ‐terpinene, potentially amplifying limonene's representation within the overall volatile profile.

Regarding fatty acid derivatives, the total losses ranged from 71.16% in the freeze‐dried sample to 93.38% in the 900 W microwave‐dried sample. Particularly, 3‐octanone, initially present at 14.76% in the fresh sample, fell below 2% in every dried variant. The contents of 1‐octen‐3‐ol and 3‐octanol remained substantially higher in freeze‐dried samples compared to other drying methods. Microwave drying was associated with the most extensive degradation of fatty acid oxidation products. These findings align with previous studies, which have consistently demonstrated that drying conditions substantially alter the volatile profile of *Thymus* species, though the direction and magnitude of these changes depend heavily on the specific method and parameters applied (Sárosi et al. 2013; Rahimmalek and Goli 2013; Venskutonis 1997). Moreover, as noted in earlier research by Venskutonis (1997), drying‐induced changes in volatile composition may vary depending on whether compounds are isolated by headspace or simultaneous distillation‐solvent extraction, due to differences in the fractions each method captures.

Microwave power level had a pronounced impact on the distribution of key volatile compound groups. As microwave intensity increased from 360 to 900 W, a progressive decrease in TMH (from 66.64% to 55.96%) and fatty acid derivatives (from 1.61% to 1.32%) were observed. This reduction can be attributed to the more rapid and intense internal heating that occurs at higher microwave powers, where localized temperatures within plant tissues may exceed the boiling point of water (Barutçu Mazı and San 2023). Such rapid thermal build‐up may accelerate volatilization and degradation of thermolabile constituents, particularly monoterpenes. Conversely, sesquiterpene hydrocarbons increased markedly (from 22.70% to 34.26%), suggesting greater thermal resilience or enhanced release due to cell matrix disruption at higher energy levels (Chua et al. 2019). Venskutonis (1997) reported that the biological structure of the oil gland trichomes of thyme was strongly affected by drying. The extent of this disruption varies depending on the drying method (Ebadi et al. 2015). Ebadi et al. (2015) reported minimal trichome damage in leaves of *Lippia citriodora* Kunth under shade and freeze drying, with damage increasing as temperature rose in oven drying. In our case, the intense internal steam pressure, generated during microwave heating, may have further increased trichome rupture, leading to enhanced permeability and diffusion of less volatile, structurally embedded compounds such as sesquiterpenes.

Across drying methods, TMH appear higher under microwave drying at 900 W (∼56%) than under freeze drying (∼52%) or shade drying (∼48%). Several concurrent factors can account for this. Although rapid internal heating during microwave drying can promote volatilization, the very short residence time and early enzyme inactivation can limit time‐dependent losses of relatively volatile monoterpenes (Orphanides et al. 2016). However, microwave treatments can also generate transient hot spots, particularly at higher power, that accelerate the thermal degradation of heat‐labile constituents. Overall, the observed outcome reflects a balance between reduced exposure time and increased thermal stress. Under freeze drying, TMH remained close to the fresh profile; observed shifts are commonly ascribed to vacuum‐assisted removal of the most volatile constituents (Chua et al. 2019; Ebadi et al. 2015; Alqarni et al. 2022). Under shade drying, TMH decreased further, consistent with residual enzymatic activity before moisture drops below activity‐limiting levels (Alqarni et al. 2022). Higher shares of fatty‐acid derivatives and, to a lesser extent, oxygenated monoterpenes in freeze‐ and shade‐dried samples could lower TMH on a percentage basis. During shade drying, increases in non‐terpenoid fractions (e.g., hexadecane) may further depress the percentage of TMH. Under oven drying, TMH may appear elevated mainly because sesquiterpenes are depleted, producing a proportional shift that concentrates relatively stable/abundant monoterpenes (e.g., limonene) in the headspace. Oven drying–induced increases in limonene have also been observed in other aromatic herbs, where it became the dominant compound in *Mentha longifolia* oil (40.8%) (Asekun et al. 2007) and was higher in *L. citriodora* compared with shade and freeze drying (Ebadi et al. 2015). In our study, the oven‐dried sample showed the lowest β‐caryophyllene (∼11% vs. ∼16%–23% with the other methods), along with lower α‐humulene, δ‐cadinene, germacrene D, β‐farnesene, and γ‐cadinene. This reduced sesquiterpene share could partly explain the relatively higher TMH after oven drying via percentage redistribution.

Previous studies on *Thymus* species have shown that drying induces compositional shifts by promoting the emergence or disappearance of specific VOCs (Chua et al. 2019). For instance, in *T. daenensis* Celak, compounds, such as α‐pinene, camphene, thymyl methyl ether, carvacrol methyl ether, and β‐selinene, emerged post‐drying (Rahimmalek and Goli 2013). In agreement with these observations, drying in our study not only caused quantitative shifts but also led to the appearance of new VOCs. Nonanal, absent in the fresh sample, appeared across all dried samples (0.32%–1.62%). This compound has been previously reported in trace amounts in the headspace volatiles of *Thymus glabrescens* Willd. (Stojanović et al. 2014). Nonanal is an aldehyde formed as a product of fatty acid oxidation. Given that thyme species are rich in oleic, linoleic, and linolenic acids, the prolonged exposure during shade drying may have contributed to elevated levels of nonanal in this treatment. Hexadecane, on the other hand, was not detected in fresh or freeze‐dried samples but appeared following other drying treatments at concentrations ranging from 0.77% to 4.49%. Hexadecane (C_16_H_34_) is a saturated hydrocarbon (*n*‐alkane). According to Kuhn et al. (2010), short‐chain, even‐numbered *n*‐alkanes, including C_16_ homologues, naturally occur in plant tissues. Therefore, the detection of hexadecane in dried samples is likely attributed to its increased extractability following cell wall rupture during dehydration (Chua et al. 2019). Compositional shifts in VOCs across drying methods reflect overlapping thermal, oxidative, and structural processes whose relative contributions vary with both processing conditions and matrix‐specific features (Venskutonis 1997; Alqarni et al. 2022). Low‐boiling monoterpenes are most susceptible to evaporative loss, whereas non‐evaporative thermal breakdown occurs when the compound itself is heat‐labile. In parallel, oxidation reactions and hydrolysis of glycosidically bound volatiles can generate new compounds during drying (Chua et al. 2019; Orphanides et al. 2016). Crucially for Lamiaceae leaves, drying can disrupt glandular trichomes and membranes, altering permeability and the partitioning of stored versus emitted constituents; the extent of damage is species and method dependent, minimal under shade and freeze drying and increases with temperature in oven/vacuum systems (Ebadi et al. 2015). Thus, the observed VOC profile alterations arise from an interplay of these effects. However, further studies are needed to establish these specific effects. Overall, the observed shifts in volatile composition reflect the combined influence of volatilization, thermal degradation, oxidation, and structural modifications, the relative contributions of which vary with the drying method, indicating that these changes follow distinct method‐dependent patterns rather than a uniform trend.

### 3.5 Principal Component Analysis

**FIGURE 5:**
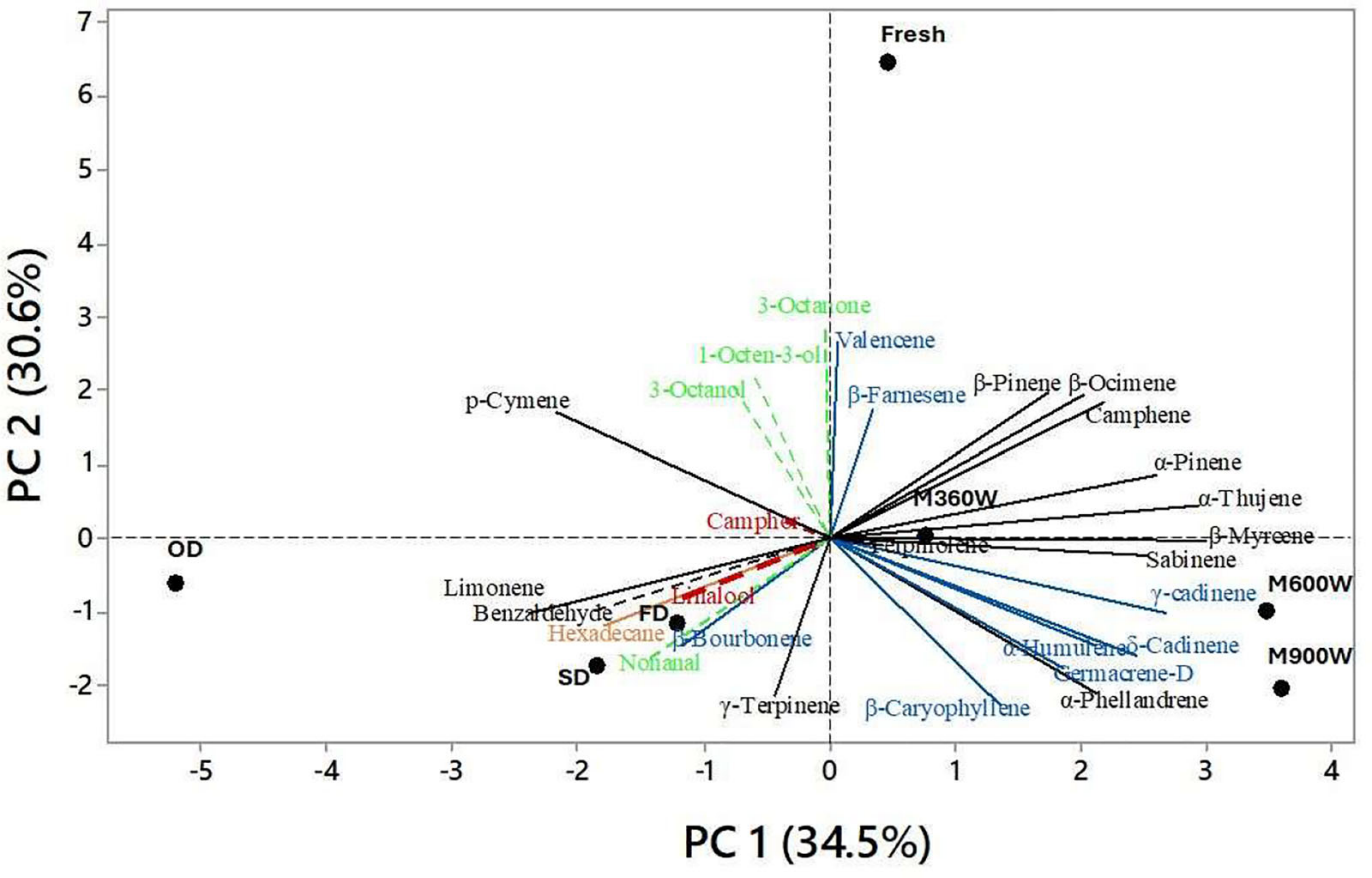
Principal component analysis (PCA) biplot illustrating the distribution of fresh and dried *Thymus praecox* subsp. *skorpilii* samples based on their headspace VOC profiles. FD, freeze‐dried; M360W, microwave‐dried at 360 W; M600W, microwave‐dried at 600 W; M900W, microwave‐dried at 900 W; OD, oven‐dried; PC1 and PC2, first two principal components; SD, shade‐dried.

PCA was employed to elucidate the similarities and differences in the VOC compositions of fresh and dried *T. praecox* subsp. *skorpilii* samples, highlighting the impact of drying methods on the volatile profile. Figure 5 presents the biplot of the first two principal components (PC1 and PC2), which together account for 65.2% of the total variance in the VOC dataset.

PC1 explains 34.5% of the variability, whereas PC2 accounts for an additional 30.7%. The distribution of the samples on the plot clearly differentiates the drying methods on the basis of their VOC profiles. VOC loadings indicate that α‐pinene (0.268), β‐caryophyllene (0.141), camphene (0.225), and β‐myrcene (0.310) are positively associated with PC1. This suggests that samples rich in these compounds, notably those subjected to microwave drying (M360W, M600W, and M900W), are aligned toward the right side of the biplot along PC1. These samples exhibited higher levels of most sesquiterpenes and the dominant monoterpene, α‐pinene. Additionally, α‐thujene exhibits a strong positive loading (0.303), further contributing to the variance explained by PC1. Conversely, limonene (−0.248) and *p*‐cymene (−0.225) showed negative loadings on PC1, suggesting that samples with elevated levels of these monoterpenes, most notably those subjected to oven drying, were positioned toward the left side of the biplot. PC2 is primarily influenced by 3‐octanone (0.328), valencene (0.310), 1‐octen‐3‐ol (0.253), 3‐octanol (0.243), β‐ocimene (0.225), β‐pinene (0.230), and camphene (0.216). In contrast, β‐caryophyllene (−0.263), γ‐terpinene (−0.249), α‐phellandrene (−0.245), and germacrene D (−0.206) contribute negatively to PC2. Importantly, the PCA results show a distinct separation between fresh and dried samples along PC2. The fresh sample is located in the upper region of the biplot, characterized by higher PC2 scores, whereas all dried samples tend to cluster below it. This clear separation further supports that each drying technique drives a distinct compositional trajectory rather than a gradual or uniform shift across samples.

## 4 Conclusion

Under the conditions of this study, different drying methods were associated with measurable changes in the TPC, color parameters, and headspace volatile profile of *T. praecox* subsp. *skorpilii*. Freeze drying provided the highest phenolic retention, whereas high‐power microwave drying (900 W) offered a favorable balance between phenolic preservation and processing efficiency due to its substantially reduced drying time. Color characteristics were similarly better preserved in freeze‐ and microwave‐dried samples, whereas oven and shade drying resulted in more pronounced darkening. Beyond individual outcomes, the findings demonstrate that drying does not lead to uniform quality changes but instead generates distinct, method‐dependent patterns across different quality attributes. These patterns are governed by the interplay between processing intensity (temperature‐time combinations), mass and heat transfer mechanisms, and matrix‐specific structural responses, including cell disruption and compound mobility. As a result, different classes of compounds exhibit divergent behaviors, with volatile profiles, phenolic content, and color responding differently to the same drying conditions.

Accordingly, the selection of an appropriate drying method should not be based on a single quality parameter but rather on the targeted product profile and processing priorities. From a broader perspective, this study provides a systematic framework for understanding drying‐induced transformations in wild thyme, emphasizing that method selection is inherently application‐driven rather than universally optimal. Future research should further elucidate the relationship between structural modifications and compound‐specific responses, particularly by integrating targeted analysis of individual phenolics and volatiles with functional quality and bioactivity assessments. Although the present study focused on relative compositional changes associated with different drying methods, absolute quantification of selected volatile constituents may provide additional insights into drying‐induced transformations.

## Author Contributions

**Didem Karadeniz**: formal analysis, data curation. **Işıl Barutçu Mazı**: conceptualization, methodology, data curation, formal analysis, writing – original draft. **Öznur Ergen Akçin**: funding acquisition, project administration, conceptualization, methodology.

## Conflicts of Interest

The authors declare no conflicts of interest.

## Data Availability

The datasets used and/or analyzed during the current study are available from the corresponding author on reasonable request.
